# Adiponectin, leptin, cortisol, neuropeptide Y and profile of mood states in athletes participating in an ultramarathon during winter: An observational study

**DOI:** 10.3389/fphys.2022.970016

**Published:** 2022-12-12

**Authors:** Camilla Kienast, Katharina Biere, Robert H. Coker, Nikolai N. Genov, Marc Jörres, Martina Anna Maggioni, Lea Mascarell-Maricic, Adriane Schalt, Magdalena Genov, Hanns-Christian Gunga, Mathias Steinach

**Affiliations:** ^1^ Charité—Universitätsmedizin Berlin, Institute of Physiology, Center for Space Medicine and Extreme Environments Berlin, Berlin, Germany; ^2^ Laboratory of Translational Research “Stress and Immunity”, Department of Anaesthesiology, University Hospital LMU Munich, Munich, Germany; ^3^ Montana Center for Work Physiology and Exercise Metabolism, University of Montana, Missoula, MT, United States; ^4^ Leap Dynamics GmbH, Berlin, Germany; ^5^ Department of Biomedical Sciences for Health, Università degli Studi di Milano, Milan, Italy; ^6^ Charité—Universitätsmedizin Berlin, Clinic for Psychiatry and Psychotherapy, Berlin, Germany

**Keywords:** ultramarathon, profile of mood states (POMS), leptin, adiponectin, NPY (neuropeptide Y), cold climate

## Abstract

**Background:** The Montane^®^ Yukon Arctic Ultra (YAU) is one of the longest (690 km) and coldest (+10.6°C–43.9°C) ultramarathons worldwide. Taking part in an ultramarathon is associated with great physiological and psychological stress, which can affect one’s mood, level of hormones, and peptides. The current study aimed to identify relationships between peptides, hormones, and mood states in participants during this ultramarathon.

**Methods:** The study cohort consisted of 36 participants (19 men, 17 women, 38.64 ± 9.12 years) split into a finisher (*n* = 10), non-finisher (*n* = 19), and control group (*n* = 7). Data were collected at four time points: baseline (PRE), during (D1 after 277 km, D2 after 383 km), and after the race (POST). Questionnaires were used to assess ratings of perceived exertion (RPE), total quality of recovery (TQR), and profile of mood states (POMS-SF). Serum NPY, leptin, adiponectin, and cortisol were measured.

**Results:** Among non-finishers, scores for confusion, anger, depression, and tension-anxiety (PRE vs. D2, *p* < 0.05) increased, while vigor decreased (PRE vs. D1, *p* < 0.05). In contrast, finishers’ tension-anxiety scores decreased (PRE vs. D1, *p* < 0.05). Fatigue increased in finishers (PRE vs. POST, *p* < 0.05) and non-finishers (PRE vs. D1, *p* < 0.05). In non-finishers, depressive mood correlated positively with leptin, anger, and confusion at several time points (*p* < 0.001). In finishers, NPY correlated with TQR at PRE (*p* < 0.05), while leptin correlated negatively with TQR at POST (*p* < 0.05). Tension-anxiety correlated highly with perceived exertion in non-finishers (*p* < 0.001) and with cortisol in finishers (*p* < 0.05) and non-finishers (*p* < 0.001). In finishers, confusion correlated negatively with NPY (*p* < 0.01).

**Conclusion:** The study reveals an essential interplay between hormones and mood states affecting performance: Leptin was associated with anger and a depressive mood state in non-finishers and worse recovery in finishers. In contrast, NPY appeared linked to a lower confusion score and heightened recovery in finishers. A simultaneous increase in depressed mood, anger, tension-anxiety, and confusion might harm performance and lead to race failure.

## 1 Introduction

In recent years, extreme sports such as ultramarathons have been gaining popularity (Scheer et al., 2020). Ultramarathons are all running distances that are longer than the classic marathon distance of 42.195 km. The spectrum of running distances in ultramarathons can last hundreds to thousands of kilometers on any terrain or surface (Scheer et al., 2020). The present study deals with the Montane^®^ Yukon Arctic Ultra (YAU), designated “the world’s coldest and toughest ultramarathon.”

Following the position statement of the Ultra Sports Science Foundation by Scheer et al. (2020), the Montane^®^ YAU can be described as follows: a race distance of 690 km, a race time limited to 14 days, belonging to the multi-day race category, the running surface is off-road mainly, and there is a total cumulative elevation gain of 6,680 m and loss of 7,001 m. The Montane^®^ YAU belongs to the self-sufficient races, meaning the athletes must carry all equipment and sustenance on their own. In particular, the participants of the Montane^®^ YAU use a sled that they pull behind them. Furthermore, a GPS tracker is attached to this sled, which sends a signal in an emergency. The Montane^®^ YAU puts the participant into an extreme situation. Lièvre and Gautier (2009) describe a situation as extreme when it has three characteristics: (1) it is evolving, (2) uncertain, and (3) risky. Similar to a polar expedition, participation in the Montane^®^ YAU requires complex and multidimensional adaptation defined by the dynamic influence of environmental and personal constraints and resources (i. e., physical and psychological) on adaptation (Nicolas et al., 2019). The Montane^®^ YAU might cause participants to push beyond their limits at the expense of their resources.

So far, there exists little quantitative data on ultramarathon runners. The number of ultramarathon participants is small, and the challenges for researchers in collecting data under sometimes extreme conditions are complicated. Besides, very few studies have examined the effects of ultramarathons in cold environments. Research shows that ultramarathons can lead to sleep and mood disturbances (Rundfeldt et al., 2018; Brager et al., 2020). In addition, there are infections, gastrointestinal symptoms, dehydration, skeletal muscle and dermatological injuries with effects on the kidneys, liver, and heart (Costa et al., 2010, 2016; Knechtle and Nikolaidis, 2018). Ultramarathon also affects the stress hormone level, increasing cortisol release (Knechtle and Nikolaidis, 2018). While most of these effects are transient, some of them can lead to severe and long-term damage.

In 2018, one of the Montane^®^ YAU participants, Roberto Zander, lost both legs and his right hand due to frostbite. Confusion due to lack of sleep and energy led to a series of detrimental decisions that eventually caused the athlete to take off his gloves and shoes and walk unprotected for 14 h, after which a search-and-rescue team found him. Especially fatigue and dehydration are predisposing factors for frostbite (McArdle et al., 2015). Dehydration and gastrointestinal problems can lead to confusion or dizziness (Glace et al., 2002). Sleep deprivation can affect cognitive performance (Hurdiel et al., 2015) and thermoregulation (Dewasmes et al., 1993). Also, acute cold exposure can impair cognitive performance (Falla et al., 2021), which might affect decision-making. This means overexposure to cold temperatures and the failure to heed warning signs in time can increase the risk of frostbite and leave permanent damage (i. e., long-term consequences). But also, dermatological injuries such as foot blisters might make the tissue more susceptible to frostbite.

Costa et al. (2016) showed that gastrointestinal symptoms and dermatological injuries (e. g., foot blisters) might lead to reduced nutritional intake, and can affect the participant’s performance and mood. Gastrointestinal symptoms were found to be negatively correlated with recovery and positively with anxiety (Urwin et al., 2021). Murray and Costa (2012) described a general perturbed immune function during an ultramarathon, which increases the risk of illness, infection, and wound healing abilities. Additional whole-body cooling affects cellular components of the immune system (Costa et al., 2010). Ultra-endurance exercise and cold exposure may evoke suboptimal host defense, increasing the risk of infection and decreasing effective wound healing.

Thusly, an ultramarathon runner has to deal with the race’s challenges and possible effects on his health, underscoring the importance of mental strength. Along this line, Schütz et al. (2012) described that “finishing an ultra race is more a matter of mind than a matter of the body.” Especially extreme situations can exacerbate stress states (Nicolas et al., 2019). Most athletes describe an increase in fatigue and a reduction in vigor (Rundfeldt et al., 2018; Graham et al., 2021). In such extreme situations, mood management seems to be of particular concern. Additionally, the survival instinct activates systems dedicated to satisfying essential needs, such as thirst, hunger, and thermoregulation. Hormones and peptides are released, acting on hypothalamic receptors to regulate energy balance, affecting behavior (e. a., increasing food-seeking) and mood (e. a., anxiety) (Kienast et al., 2019). The hypothalamus releases the orexigenic peptide neuropeptide Y (NPY) in response to stress (e. g., cold exposure, exercise), which might strengthen mental and physical performance (Kienast et al., 2019).

Research shows that during endurance exercise, adiponectin and NPY levels increase (Karamouzis et al., 2002; Czajkowska et al., 2020). By contrast, the counterplayer of NPY, the adipocyte-derived hormone leptin, decreases (Karamouzis et al., 2002). Leptin belongs to the anorexigenic hormones (Kienast et al., 2019). These peptides and hormones, regulating energy expenditure and food intake, might exhibit a specific interplay of unknown importance affecting mood states in ultramarathon athletes.

Therefore, the current study aims (1) to identify changes in the profile of mood states, (2) and to examine the relationships between hormonal/neurotransmitter variables (adiponectin, leptin, cortisol, and NPY) and different mood states during an Arctic ultramarathon. On the one hand, the study’s design permits hormonal and neuropeptide assessment during a stable low-stress and high-stress phase, ensuring a uniform observation of various stressors across participants. On the other hand, the Montane^®^ YAU provides an ethologically realistic model of acute, uncontrollable stress that closely approximates stress that military and astronauts may perceive during similar occupational conditions.

## 2 Materials and methods

### 2.1 The race

The Montane^®^ YAU is an ultramarathon in the extreme environment of Canada’s Arctic north-western Yukon Territory. The race occurs during February each year with different distances, where the longest distance of 690 km (430 miles) takes place only every 2 years. The race starts in Whitehorse and finishes at Dawson City. The trail is prepared and marked with wooden sticks with fluorescent tops. The surface is uneven and snow-covered, which can be knee-deep and hard to travel. The first part of the trail goes onto the frozen Yukon River, then over watersheds and several gradual, sometimes steep climbs. Overflow can be slowly rising water, possibly snow-covered, which may be difficult to navigate since the surface is not flat and very slippery. Among moose and bison, the athletes can also encounter wolves. After the first 160 km, the trail frequently goes on open fields and marsh, followed by a tight bush trail through the forest with parts of fallen trees on the path. The trail crosses several frozen lakes, which can have “soft spots” where the athletes can break through and end up in the water. The last part of the trail goes on the frozen Klondike River into Dawson City.

At the beginning of the race, the altitude is 610 m in Whitehorse, goes up to 1157 m at the highest point (King Solomon Dome), and ends at 319 m in Dawson City.

The duration of the race is limited to 14 days and must be completed during this time. This represents a minimum daily running distance of 49.28 km, more than one marathon (42.195 km) daily. The race has ten checkpoints that athletes should reach in a certain time. However, it is also possible not to reach a stage in the given time and to make up the time until the next stage. The ten checkpoints (stages) are in the following order: (1) Muktuk Adventures (42.195 km), (2) Dog Grave Lake (95 km), (3) Braeburn (160 km), (4) Mandanna Lake (235 km), (5) Carmacks (277 km), (6) McCabe (340 km), (7) Pelly Crossing (383), (8) Pelly Farm (438 km), (9) Pelly Crossing (300-mile finish) (483 km), and (10) Scroggie Creek (542 km). At each checkpoint, medical examinations are conducted by a medical team (including a medical doctor). The athletes are particularly examined for frostbite, in which case they are immediately excluded from the race. Each athlete can get one hot meal and hot water at the checkpoints; otherwise, the race is self-sufficient. The athletes must carry their survival equipment, tent, clothes, and food independently, using a carriage sled that they pull behind while walking (accounting for 20–30 kg). Running out of food between checkpoints is a reason for disqualification. The athletes are allowed to store and change up to three bags at the checkpoints. Except for two checkpoints (Carmacks at 277 km and Pelly Crossing at 383 km), athletes sleep and perform all activities outside.

For safety regulations, each athlete has to participate in survival training the week before the race. The training course includes the preparation for the extremely demanding conditions. For example, athletes must prove they can change clothes when wet or make a fire in a certain amount of time to stay warm and melt snow. For the race, special gear is mandatory (e. a., an avalanche shovel). Forgetting mandatory gear may result in a time penalty of up to 12 h.

The athletes are tracked with a GPS device (Spot^®^, Spot LLC, Virginia, United States) that is mounted on a carriage sled or pulk. In case of an emergency, they can use it to call for assistance. When athletes start to bivvy (sleep), they must push the “Custom Message Button” on their Spot to inform the race organizers. It is not allowed to sit on their sleds on the mountain descent. Any acceleration is registered by the Spot and can lead to disqualification.

**FIGURE 1:**
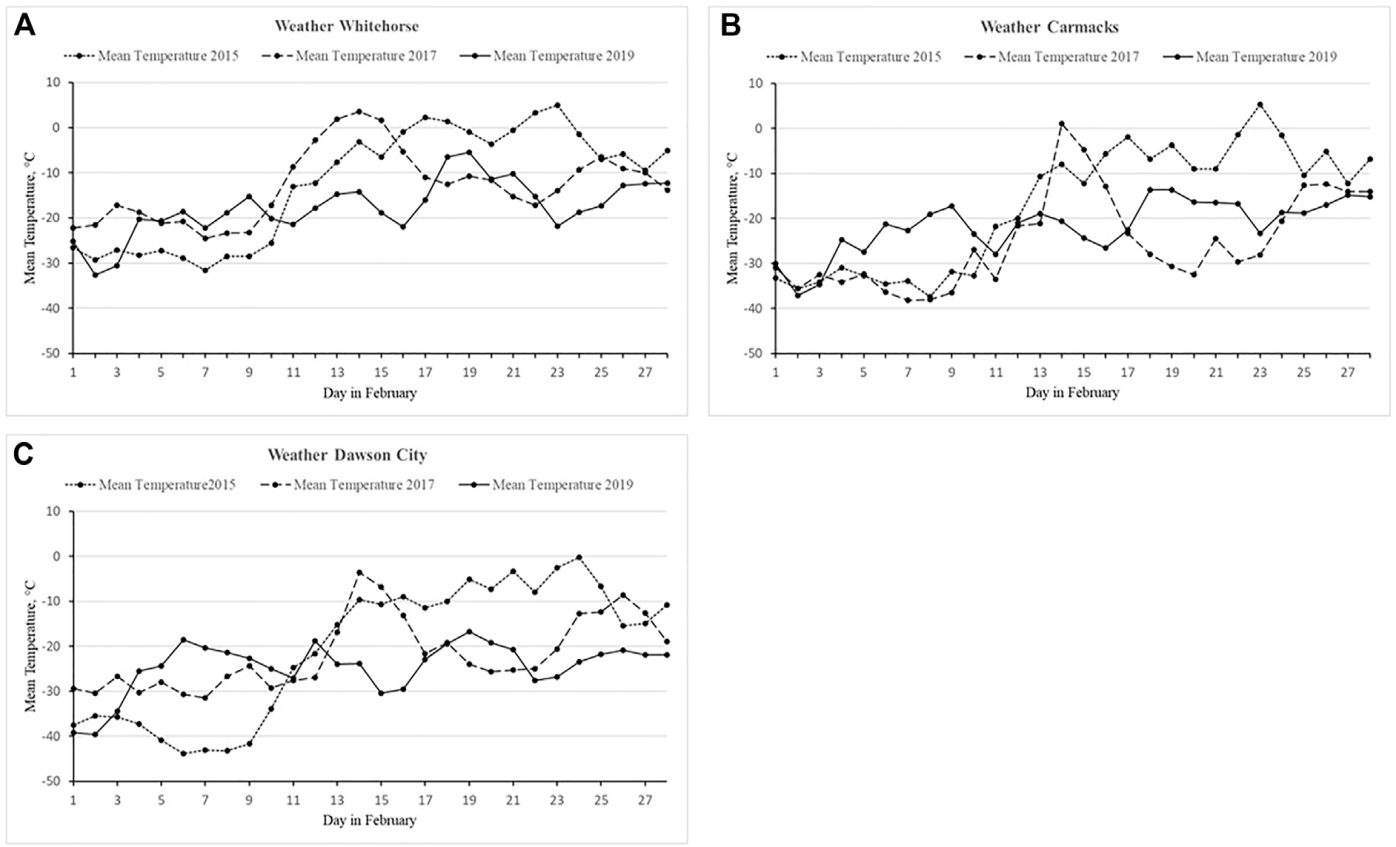
Weather conditions across editions. Mean air temperature was measured in Whitehorse **(A)**, Carmacks **(B)** and Dawson City **(C)** in February 2015 (dotted line), February 2017 (long dashed line), and February 2019 (solid line). No significant differences between editions or locations.

The athletes are exposed to adverse weather conditions, including hail and snow storms. Air temperatures recorded at the closest meteorological stations within the course during racing ranged from + 10.6°C and – 43.9 C (Figure 1) (weather data of the Government of Canada, 2020). More detailed information about the race is provided on the official website of the Montane^®^ YAU (https://arcticultra.de/).

### 2.2 Subjects and study implementation

**FIGURE 2:**
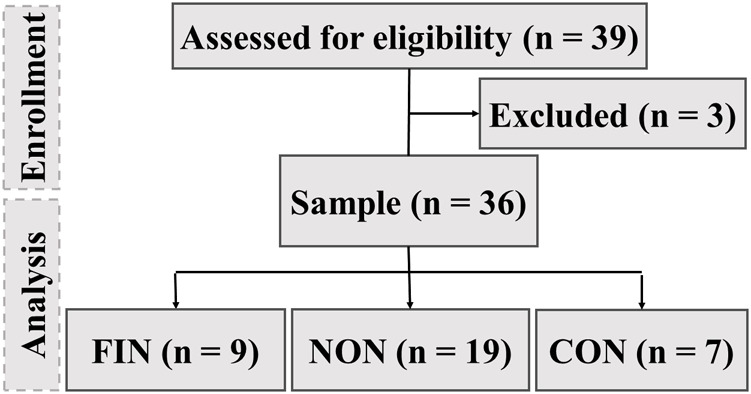
Overview of recruitment procedure. FIN = Finisher, NON = Non-finisher, CON = Control group.

This study is part of continuing research since 2013: “Physiological changes of participants of the Yukon Arctic Ultra - an ultramarathon in an extremely cold climate”. In our previous analyses, we evaluated (1) the metabolic responses of participants in the YAU (Coker et al., 2017), (2) the influence of energy balance on fat-free mass (Schalt et al., 2018), (3) cardiac autonomic modulations in conjunction with psychological correlates (Rundfeldt et al., 2018), and influences on glycocalyx shedding during ultra-endurance exercise (Steinach et al., 2022). In 2019, we continued our investigation of athletes participating in the 690-km race. This is an observational study because ultramarathon is a difficult-to-analyze topic, and study participants could not be randomized efficiently. Generating a large sample size is highly challenging because few people participate in an ultramarathon under such extreme conditions. To further strengthen the significance of our analysis, we combined the number of participants from 2013, 2015, and 2019 (*n* = 32). We included a control group (*n* = 7) based on the on-site volunteers for the first time. During the years 2015, 2017, and 2019, 32 athletes (13 female, 19 male) enrolled in the study: nine in 2015 (4 female, five male), 10 in 2017 (2 female, eight male), and 13 in 2019 (7 female, six male). In 2019, 7 (5 female, two male) volunteers not partaking in the 690-km race served as a control group. Of the 32 participants and the seven control (*n* = 39), only 36 (19 men, 17 women) were included in the data analysis (Figure 2).

Compared to the athletes, the volunteers in the control group were moderately active. They traveled by car or snowmobile up north towards Dawson City and took care of the preparation of the route. Their main tasks were maintenance of the route markings, luggage packing, and the support team’s catering (food preparation). Compared to the athletes, they spent less time outside and, thus, less exposed to cold weather conditions. They only slept indoors.

The recruitment for this study was conducted with the support of the event organizers. A call for participants, with a brief description of the research and planned measurements, was transmitted to the athletes. The organizers were encouraged to contact experienced athletes who had already participated in ultramarathons. Since such a study is difficult to reproduce, it was advantageous at this point to include already experienced athletes. Athletes and volunteers interested in the study contacted the principal investigator *via* e-mail and received further detailed information. The potential study participants had several weeks to ask questions *via* e-mail and decide whether to participate in the study. There were no further inclusion or exclusion criteria: all 690-km race category athletes were eligible to enter the study. This procedure was necessary because, due to the small number of participants in such races, it would otherwise have been difficult to gather sufficient study participants. All athletes were required to present a health certificate issued by their home physician to the event organizers to partake in the race. During a meeting in Whitehorse, Yukon Territory, Canada, 4–5 days before the race started, the potential study participants met with the investigators in person, had the chance to ask further questions, and finally gave their informed written consent to partake in the study. The Charité Universitätsmedizin Berlin Ethic Committee approved the study (review number EA4/109/12), all measurements and procedures complied with the Declaration of Helsinki (6th Revision 2008; Korea) regarding the treatment of human subjects.

### 2.3 Experimental protocol and measurements

**FIGURE 3:**
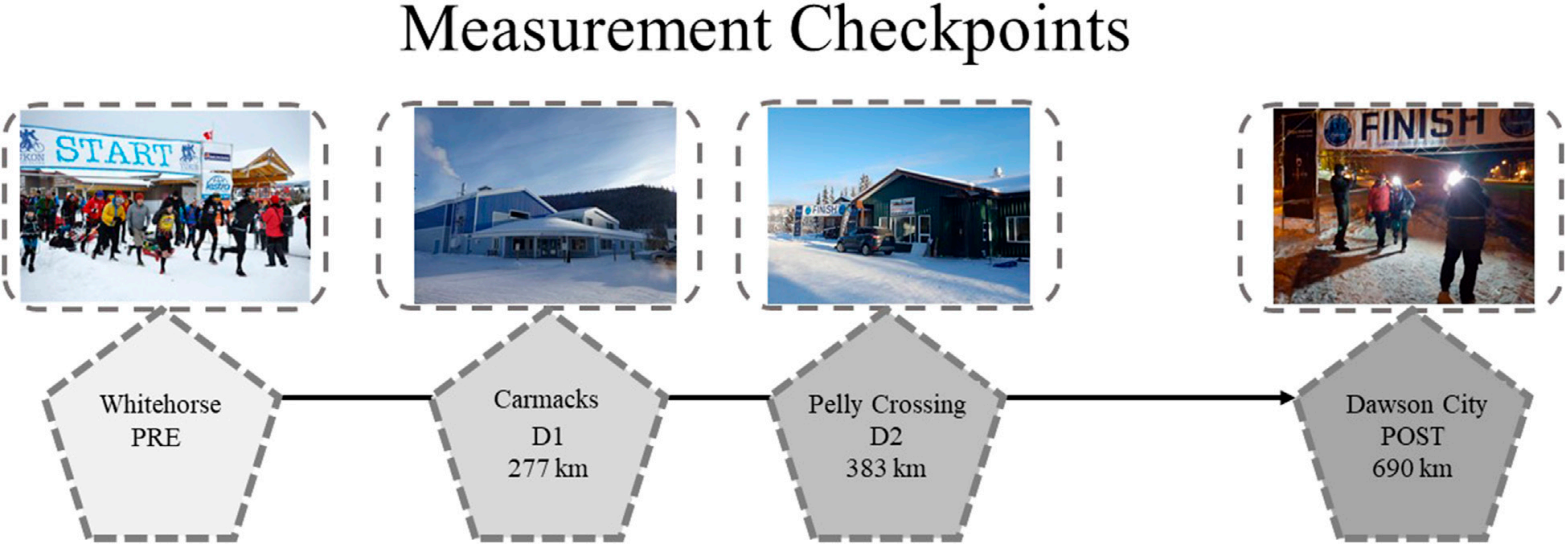
The measurement checkpoints: before the race in Whitehorse (PRE), during the race in Carmacks at 277 km (D1), and Pelly Crossing at 383 km (D2), after the race in Dawson City at 690 km (POST).

The measurements were performed before and after the race and at two in-race checkpoints: (1) before the start of the race in Whitehorse (PRE), (2) at the Carmacks in-race checkpoint at 277 km (During 1, D1), (3) at the Pelly Crossing in-race checkpoint at 383 km (During 2, D2) and (4) immediately after completion of the race in Dawson City at 690 km (POST) (Figure 3)*.* The measurement checkpoints were all indoors and were chosen for accessibility, available electricity, water supply, sufficient space, comfortable ambient temperature, and low noise to perform all measurements and the blood collection under controlled conditions. The GPS data of the Spot were used to access the velocity of each athlete for further analysis (Table 2).

### 2.4 Anthropometric data

Anthropometric data were collected at the four measurement points after rest. Height was measured by the participants prior to the race and collected *via* e-mail by the study scientist. The body weight was measured at each measurement point with a calibrated scale (Seca, Germany, accuracy +/−100 g) on an even surface, and the participants were dressed in light underwear. Afterward, the body mass index (BMI) was calculated from these two parameters for each measurement point. In addition, body composition was determined by bioelectrical impedance analysis (BIA) using an Akern BIA 101 after rest, when the bladder was empty, and before breakfast. BIA is an established method to determine body composition that our department has already used in other studies (Schalt et al., 2018; Jörres et al., 2021). The BIA measurements provided values for fat mass using equations appropriate for this cohort, based on Segal et al. (1988) (Segal et al., 1988) and Sun et al. (2003) (Sun et al., 2003) calculation methods.

### 2.5 Blood parameters

Blood was taken at each measurement checkpoint after rest and before food intake. In order to achieve the required amount of serum volume, 15 ml of whole blood were drawn from the cubital vein into a serum tube (Sarstedt S-Monovette^®^, 50 units/ml) and immediately stored in a refrigerating unit at +2 to +8°C. Afterward, the samples were centrifuged (10 min at 2,000 x g) to separate serum from the cellular compounds, and the serum was pipetted into cryovials. Finally, the samples were stored in a liquid nitrogen Dewar for transport (−196°C). Without interrupting the cold chain, the serum samples were transported to Berlin. In the clinical and certified laboratory in Berlin (Germany) [Labor 28 GmbH, Berlin, Germany, accredited at the “DAkkS” (Deutsche Akkreditierungsstelle GmbH), the national accreditation body for the Federal Republic of Germany, according to regulation (EC) number 765/2008 and the accreditation body act of Germany] the samples were thawed to analyze cortisol (μg/dl), adiponectin (μg/dl), and leptin (ng/ml). The Ludwig-Maximilians-University of Munich (Germany) laboratory conducted further serum analyses for NPY (pg/ml).

### 2.6 Psychometric assessment

At each measurement checkpoint, a psychometric measurement was assessed. Standardized questionnaires were used to rate perceived exertion directly after arrival at the checkpoints (before rest) and the quality of recovery after rest. The assessment of mood states was conducted after rest.

#### 2.6.1 Profile of mood states - Short form

Mood states were measured using the Shacham (1983) shortened ‘right now’ version of the Profile of Mood States (POMS) questionnaire (Shacham, 1983). The test requires the participant to indicate the state of emotions experienced in the past hours. The POMS-SF contains 37 single-word mood descriptors, each with a 5-point Likert response scale, from which six mood subscale scores for tension-anxiety, depression-dejection, anger-hostility, vigor-activity, fatigue-inertia, and confusion-bewilderment could be calculated. Finally, the test yields five negative mood states (tension-anxiety, depression, anger, fatigue, confusion) and one positive mood state (vigor) (Shacham, 1983). To minimize response bias effects, participants were briefed to complete the POMS-SF based on how they felt now rather than attempting to memorize their previous mood. Every time a participant completed a POMS-SF, they could not see any previous questionnaire they had completed before.

#### 2.6.2 Borg ratings of perceived exertion

Ratings of Perceived Exertion is a one-item self-report measure of perceived physical exertion. The content of this scale is a numerical indication of 6–20 of exertion. A score of six is comparable to a resting activity, and a score of 20 characterizes exhaustive exercise (Borg, 1962, Borg, 1970).

#### 2.6.3 Borg total quality of recovery

As well as the perceived exertion scale, the Total Quality of Recovery scale contains a numerical indication of 6–20. By contrast, the total quality of recovery monitors the recovery process. A score of six means a very, very poor recovery and 20 a very, very good recovery (Kenttä and Hassmén, 1998).

## 3 Statistics

### 3.1 Data analysis and software used

The data were analyzed with R version 4.0.3 and R Studio 1.3.1093. The visualizations were created with the ggplot2 R package and with the support of the ggpubr R package for the addition of statistical tests. The statistical testing between the groups was performed with the t-test for two groups, e. g., finisher vs. non-finisher. The paired t-test was used to compare the effect of the same values in one group at different measurement points, e.g., PRE vs. POST. The correlation analysis and visualization were performed with the PerformanceAnalytics R package. The correlation analyses were conducted to find a relationship between different attributes in the three subgroups overall measurement points. Statistically, Pearson’s co-efficient correlation was quantified. Peak detection in the data was performed using the R package pracma, with a minimum of seven increasing steps before reaching a peak set as the parameter for the ‘findpeaks’ function. A minimum peak spacing of 50 was used. Statistical summary tables containing arithmetic mean ± and standard deviations (m ± SD) values were created with the qwraps2 R package. The scales R package was used to work with the timestamps included in the data. The results of the statistical tests were considered significant if a *p*-value was below 0.05.

## 4 Results

### 4.1 Anthropometric characteristics

Participants were divided into three groups, which were also split by sex (Table 1). The three groups consisted of the finishers, non-finishers, and a control group. The finishers completed a total distance of 690 km. Those who did not finish the race gave up at an earlier stage of the race, or they were excluded from the race by the organizers for health reasons (e. g., frostbite) or because they were too slow. Too slow, in this case, meant that the organizers could estimate in what time span an athlete should be at one of the first checkpoints based on experience from previous races. In this case, the athlete was taken to the next checkpoint with a snowmobile. This allowed our study team to investigate these athletes as well directly after they were excluded from the race. The 690-km ultramarathon was completed by nine of 29 entrants, 20 withdrew from the competition at earlier points. Most of the study participants were of Caucasian descent, and one was of Asian origin. Their anthropometric data are presented in Table 1. There were no significant differences between the groups neither between women and men regarding age. The mean age of all participants was 38.64 (± 9.1). Women had lower weight and height (*p* < 0.001). They exhibited a higher fat mass (*p* < 0.001), except for the finisher group. In finishers were no differences in weight and height between the sexes. The BMI did not differ between the sexes across all groups.

**TABLE 1:** Subject demographics at baseline for all participants and in subgroups. FIN = Finisher, NON = Non-finisher, CON = Control group, FM = fat mass, BMI = body mass index, m = mean, SD = Standard Deviation.

**Parameter**	**All (n = 36)**		**All athletes (n = 29)**		**FIN (n = 10)**		**NON (n = 19)**		**CON (n = 7)**	
		**m ± SD**		**m ± SD**		**m ± SD**		**m ± SD**		**m ± SD**
**Age (years)**										
Men	38.32	9.29	38.71	9.45	42.60	11.59	37.08	8.45	35.00	9.90
Women	39.00	9.20	40.00	7.89	37.40	6.19	41.86	8.88	36.60	12.54
All	38.64	9.12	39.24	8.71	40.00	9.18	38.84	8.69	36.14	11.04
p-value (women vs. men)	0.826		0.701		0.548		0.259		0.880	
**Weight (kg)**										
Men	79.61	9.16	79.89	9.63	77.08	8.88	81.07	10.06	77.20	3.68
Women	64.97	9.76	65.57	11.37	64.40	10.34	66.41	12.79	63.52	4.69
All	72.70	11.90	73.97	12.46	70.74	11.28	75.67	13.01	67.43	7.84
p-value (women vs men)	<0.001		0.001		0.095		0.013		0.015	
**Height (cm)**										
Men	178.84	7.01	177.82	6.69	175.40	6.54	178.83	6.77	187.50	0.71
Women	167.94	8.00	167.33	7.94	168.60	6.58	166.43	9.18	169.40	8.88
All	173.69	9.22	173.48	8.83	172.00	7.15	174.26	9.69	174.57	11.43
p-value (women vs. men)	<0.001		0.001		0.140		0.007		0.042	
**FM (%)**										
Men	16.37	2.67	16.79	2.48	18.05	2.63	16.26	2.32	12.83	1.36
Women	25.04	4.41	24.95	4.94	24.15	3.92	25.52	5.79	25.25	3.24
All	20.46	5.64	20.17	5.46	21.10	4.50	19.67	5.96	21.70	6.64
p-value (women vs. men)	<0.001		<0.001		0.020		<0.001		0.004	
**BMI (kg/m²)**										
Men	24.90	2.57	25.24	2.47	25.10	3.09	25.30	2.33	21.96	1.21
Women	23.17	4.08	23.53	4.50	22.60	2.80	24.20	5.54	22.30	3.08
All	24.08	3.43	24.53	3.49	23.85	3.08	24.89	3.72	22.21	2.57
p-value (women vs. men)	0.133		0.199		0.217		0.526		0.891	

### 4.2 Race performance

The average time to complete the course was 264:13 (± 25:30) hours. Men took less time to complete the race with 249:28 (± 22:51) hrs:min than women, who took 278:58 (± 19:57) hrs:min (Table 2). In finishers, the moving average velocity was 4.07 (± 0.68) km/h, whereas, in non-finishers, the overall moving velocity was 4.14 (± 0.56) km/h (Table 2). The non-finishers reached distances between 53.11 and 643.73 km. Thus, some participants from the non-finisher group (*n* = 3) were also examined at the study endpoint (POST).

**TABLE 2:** Women vs. men’s performance data for finishers and non-finishers. The overall completed distance, finishing time, and the total and moving average velocity. FIN = Finisher, NON = Non-finisher, m = mean, SD = Standard Deviation.

**Parameter**	**All athletes (n = 29)**		**FIN (n = 10)**		**NON (n = 19)**	
		**m ± SD**		**m ± SD**		**m ± SD**
**Distance (km)**						
Men	367.32	237.34	690.00	0.00	232.86	122.00
Women	472.85	239.69	690.00	0.00	317.38	194.83
All	410.98	239.91	690.00	0.00	264.13	153.35
p-value (women vs. men)	0.278		1.000		0.256	
**Finishing Time (hrs:min)**						
Men	143:23	89:45	249:28	22:51	95:10	60:43
Women	198:28	100:56	278:58	19:57	131:23	90:25
All	165:49	96:34	264:13	25:30	107:57	71:57
p-value (women vs. men)	0.098		0.062		0.451	
**Total Average Velocity (km/h)**						
Men	2.89	0.62	2.79	0.34	2.93	0.72
Women	2.43	0.20	2.36	0.16	2.48	0.22
All	2.70	0.54	2.58	0.34	2.76	0.62
p-value (women vs. men)	0.023		0.031		0.128	
**Moving Average Velocity (km/h)**						
Men	4.45	0.44	4.57	0.29	4.39	0.50
Women	3.69	0.49	3.57	0.59	3.77	0.44
All	4.11	0.60	4.07	0.68	4.14	0.56
p-value (women vs. men)	<0.001		0.009		0.018	

### 4.3 Blood parameters

Leptin, NPY, adiponectin, and cortisol levels are depicted in the Supplementary Tables.

### 4.4 Profile of mood states—short form

Supplementary Figure S1 shows the six mood dimensions of the POMS-SF in the three groups at the four measurement points. For a simplified presentation and description of the results, the six dimensions of mood are written out in abbreviated form with the first word before the hyphen except for tension-anxiety. Tension-anxiety scores were significantly higher in all athletes than in the control group at baseline (t-test, *p* < 0.05). The non-finishers had higher tension-anxiety scores than the finishers at D1 (t-test, *p* < 0.05). And tension-anxiety increased in non-finishers (paired t-test, PRE vs. D2 *p* < 0.05). In contrast, tension-anxiety decreased in finishers throughout the race (paired t-test, PRE vs. D1, PRE vs. D2, and PRE vs. POST *p* < 0.05). At D2, non-finishers had a significantly higher depressive mood score than the control group (t-test, *p* < 0.05). The level of depressive mood score increased significantly PRE vs. D1 in non-finishers (paired t-test, *p* < 0.05) but not in finishers. We found no significant difference in anger scores between the three groups, even though anger increased in non-finishers significantly during the race (paired t-test, PRE vs. D1, *p* < 0.05). There was no significant difference in vigor at baseline between the three groups. After the race (POST), the level of vigor was higher in the finisher group than in the control group (t-test, *p* < 0.05). In non-finishers and finishers, vigor decreased. But only in non-finishers the reduction in vigor scores was significant (paired t-test, PRE vs. D1, *p* < 0.05). Across the race, fatigue increased in finishers (paired t-test, PRE vs. D1, *p* < 0.05, PRE vs. D2, *p* < 0.001, PRE vs. POST, *p* < 0.05) and in non-finishers (paired t-test, PRE vs. D1, *p* < 0.05). No significant difference was found between non-finishers and finishers in fatigue scores. Before the start of the race, confusion scores were rated higher in all athletes together vs. the control group (t-test, D1 *p* < 0.05). But there was no significant difference between the three groups at baseline, during and after the race. In non-finishers, confusion increased during the race (paired t-test, PRE vs. D1, *p* < 0.05) but not in finishers.

### 4.5 Correlation analyses

**FIGURE 4:**
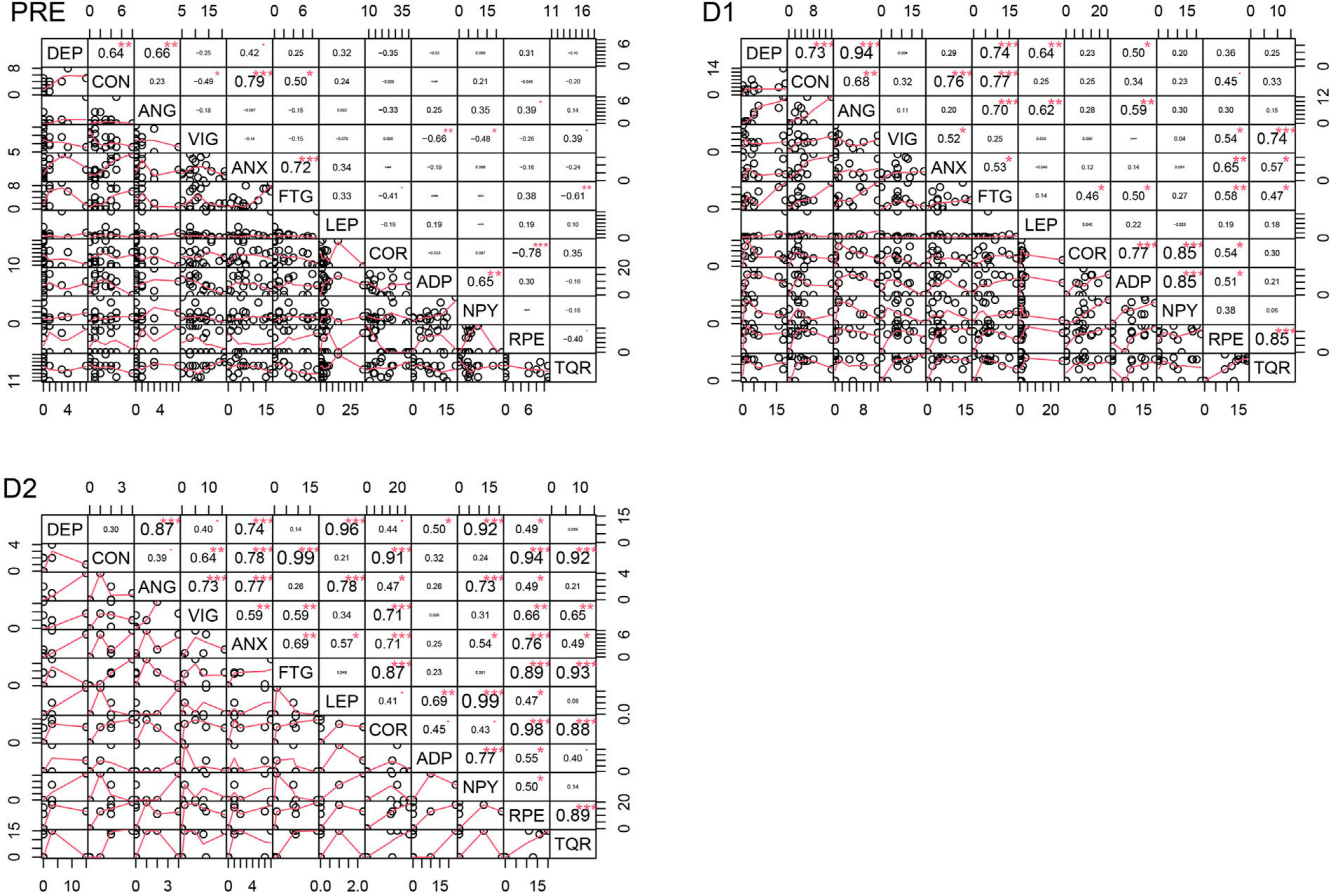
Correlation analyses for the non-finisher group at the four time points. DEP = Depression, CON = Confusion, ANG = Anger, VIG = Vigor, ANX = Tension-ANXiety, FTG = Fatigue, LEP = Leptin, COR = Cortisol, ADP = Adiponectin, NPY = Neuropeptide Y, RPE = Ratings of Perceived Exertion, TQR = Total Quality of Recovery.

**FIGURE 5:**
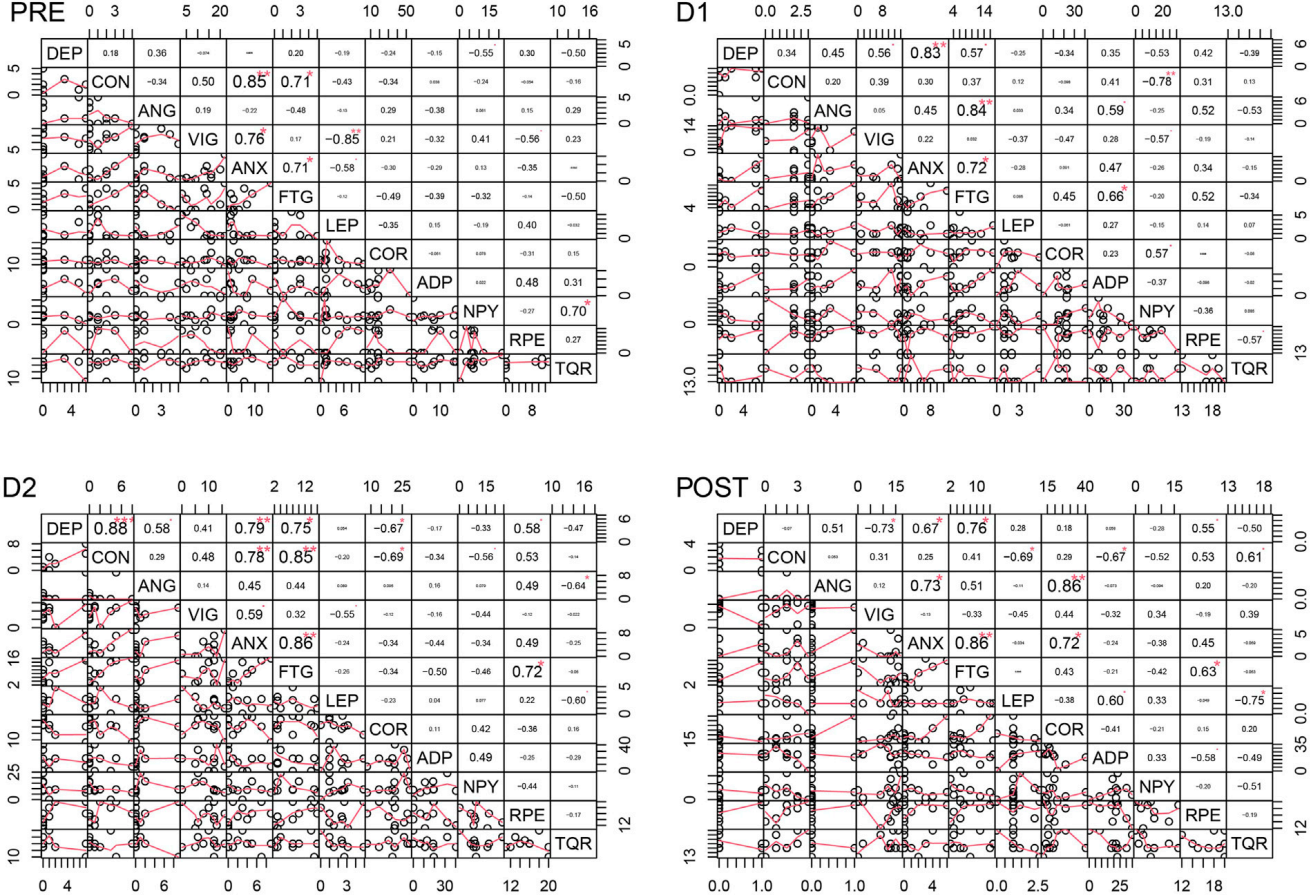
Correlation analyses for the finisher group at four time points. DEP = Depression, CON = Confusion, ANG = Anger, VIG = Vigor, ANX = Tension-ANXiety, FTG = Fatigue, LEP = Leptin, COR = Cortisol, ADP = Adiponectin, NPY = Neuropeptide Y, RPE = Ratings of Perceived Exertion, TQR = Total Quality of Recovery.

**FIGURE 6:**
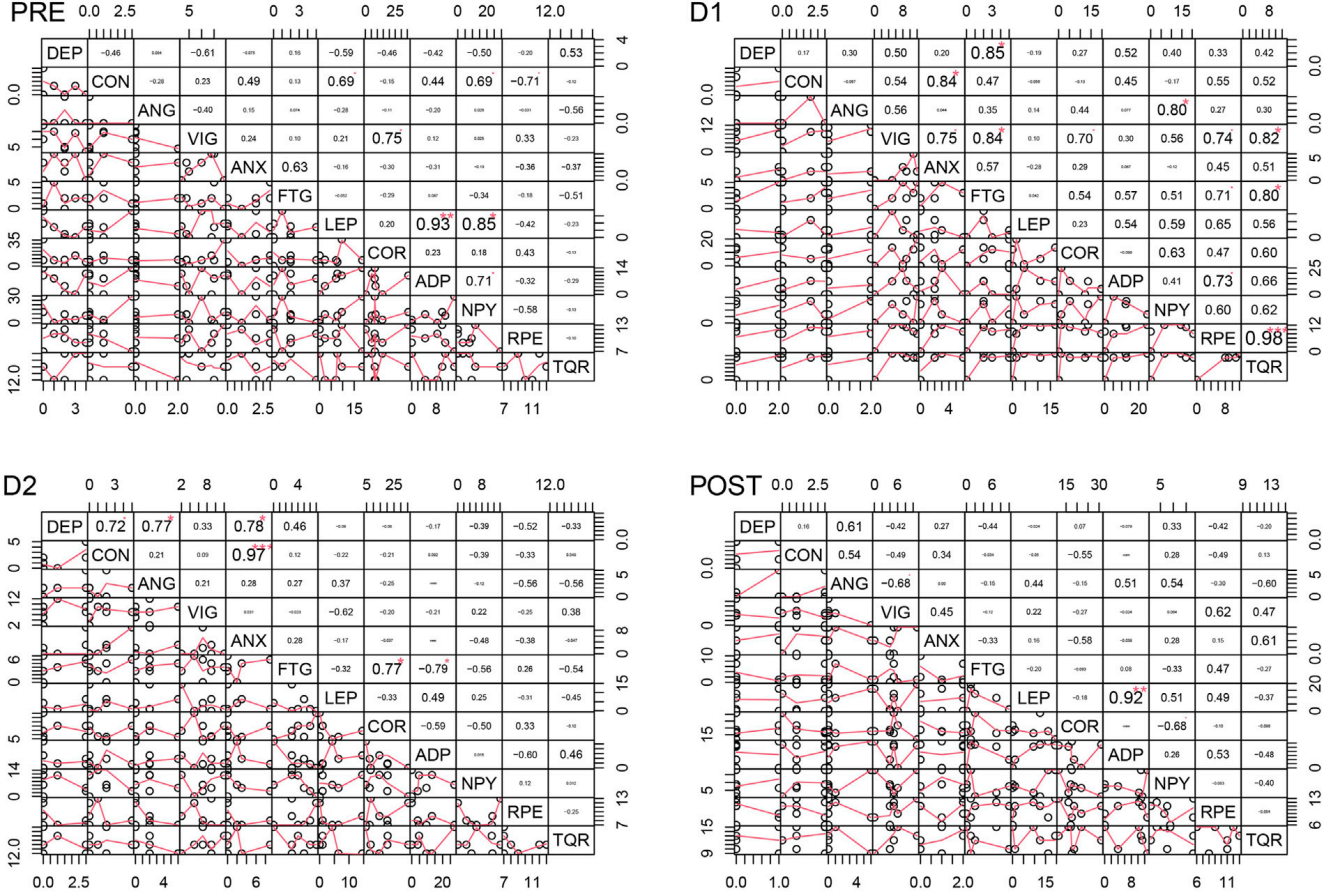
Correlation analyses for the control group at the four time points. DEP = Depression, CON = Confusion, ANG = Anger, VIG = Vigor, ANX = Tension-ANXiety, FTG = Fatigue, LEP = Leptin, COR = Cortisol, ADP = Adiponectin, NPY = Neuropeptide Y, RPE = Ratings of Perceived Exertion, TQR = Total Quality of Recovery.

Figures 4–6 depict the correlation analysis results of the psychometric assessment, BORG scales, peptides and hormones. Figure 4 shows the result of the correlation analyses for non-finishers and Figure 5 for finishers. Figure 6 shows the result of the control group. In the non-finisher group, the data collected at the POST measurement point were not sufficient to perform a correlation analysis. Therefore, in Figure 4, the POST measurement point is not shown as it can be found in the figures and tables of the other values. A wide variety of correlations between the peptides and hormones were emerging. Of interest are the significantly lower correlations in the control group compared to the athlete subgroups. At baseline, leptin correlated positively with adiponectin (PRE Pearson 0.93, *p* < 0.01) and NPY (PRE Pearson 0.85, *p* < 0.05) in the control group. In the non-finisher group, this positive correlation was also found, but by the end of the race (D2 Pearson 0.69, *p* < 0.01). At D1, cortisol correlated positively with NPY in finishers (Pearson 0.57, *p* < 0.05), and in non-finishers with adiponectin (Pearson 0.77, *p* < 0.01) and NPY (Pearson 0.85, *p* < 0.001). There was no correlation between cortisol, NPY and adiponectin at any measurement point in the control group. In the control group, adiponectin correlated positively with NPY (PRE Pearson 0.71, *p* < 0.05). There was an apparent correlation between adiponectin and NPY, at all time points in non-finishers (PRE Pearson 0.65, *p* < 0.01; D1 Pearson 0.85, *p* < 0.001; D2 Pearson 0.77, *p* < 0.001). In contrast, this correlation was missing in finishers.

For a better overview, other results of the correlation analysis are described starting with one of each of the six mood dimensions. Only significant results are described in this section.

#### 4.5.1 Tension-anxiety

In non-finishers and finishers, tension-anxiety correlated with fatigue at all measurement points (Non-finishers: PRE Pearson 0.72, *p* < 0.001; D1 Pearson 0.53, *p* < 0.05; D2 Pearson 0.69, *p* < 0.01 and finishers: PRE Pearson 0.71, *p* < 0.05; D1 Pearson 0.72, *p* < 0.05; D2 Pearson 0.86, *p* < 0.01; POST Pearson 0.86, *p* < 0.01). This correlation was highly significant in non-finishers at baseline. By contrast, this correlation was not found in the control group.

In non-finishers, tension-anxiety correlated high with perceived exertion (D1 Pearson 0.65, *p* < 0.01; D2 Pearson 0.76, *p* < 0.001) and low with total quality of recovery (D1 Pearson 0.57, *p* < 0.05; D2 Pearson 0.49, *p* < 0.05). Tension-anxiety correlated positively with cortisol in finishers (POST Pearson 0.72, *p* < 0.05) and non-finishers (D2 Pearson 0.71, *p* < 0.001).

#### 4.5.2 Depressive mood

At baseline and during the first part of the race, the depressive mood score was positively correlated with confusion in non-finishers (PRE Pearson 0.64, *p* < 0.01; D1 Pearson 0.73, *p* < 0.001). This correlation was only found at D2 and not at the other measurement points in finishers (D2 Pearson 0.88, *p* < 0.001) and the control group (D2 Pearson 0.72, *p* < 0.05). Furthermore, the depressive mood score correlated positively with anger in non-finishers at each measurement point (PRE Pearson 0.66, *p* < 0.01; D1 Pearson 0.94, *p* < 0.001; D2 Pearson 0.87, *p* < 0.001) and in the control group at D2 (D2 Pearson 0.77, *p* < 0.05). The depressive mood score correlated positively with tension-anxiety (Non-finishers: D2 Pearson 0.74, *p* < 0.001; Finishers: D1 Pearson 0.83, *p* < 0.01; D2 Pearson 0.79, *p* < 0.01; POST Pearson 0.67, *p* < 0.05; Control group: D2 Pearson 0.78, *p* < 0.05), and fatigue in all groups (Non-finishers: D1 Pearson 0.74, *p* < 0.001; Finishers: D1 Pearson 0.57, *p* < 0.05, D2 Pearson 0.75, *p* < 0.05; Control group: D1 Pearson 0.85, *p* < 0.05). The relationship between the depressive mood score, tension-anxiety, and fatigue was highly significant in non-finishers. There was a positive correlation between the depressive mood score and leptin at D1 (Pearson 0.64, *p* < 0.01) and D2 (Pearson 0.96, *p* < 0.001) in non-finishers. But also between the depressive mood score and adiponectin (Non-finishers: D1 Pearson 0.50, *p* < 0.05) and NPY (Non-finishers: D2 Pearson 0.92, *p* < 0.001). The depressive mood score correlated negatively with cortisol at D2 in finishers (Pearson -0.67, *p* < 0.05). These findings were not present in other groups.

#### 4.5.3 Anger

In finishers and non-finishers, anger correlated positively with fatigue (Non-finishers: D1 Pearson 0.70, *p* < 0.001; Finishers: D1 Pearson 0.84, *p* < 0.01) and tension-anxiety (Non-finishers: D2 Pearson 0.77, *p* < 0.001; Finishers: POST Pearson 0.73, *p* < 0.05). In finishers, anger correlated positively with cortisol (POST Pearson 0.86, *p* < 0.01), and was also negatively correlated with total quality of recovery (D2 Pearson −0.64, *p* < 0.05). In the control and non-finisher groups, anger correlated positively with NPY (Non-finishers: D2 Pearson 0.73, *p* < 0.01; Control group: D1 Pearson 0.80, *p* < 0.05). In non-finishers also with leptin (D1 Pearson 0.62, *p* < 0.01; D2 Pearson 0.78, *p* < 0.001), adiponectin (D1 Pearson 0.59, *p* < 0.01), cortisol (D2 Pearson 0.47, *p* < 0.05), perceived exertion (D2 Pearson 0.49, *p* < 0.05) and vigor (D2 Pearson 0.73, *p* < 0.001).

#### 4.5.4 Vigor

Vigor was positively correlated with total quality of recovery in non-finishers (PRE Pearson 0.39, *p* < 0.05, D1 Pearson 0.74, *p* < 0.001, D2 Pearson 0.65, *p* < 0.01) and the control group (D1 Pearson 0.82, *p* < 0.05). By contrast, in finishers, it was NPY that correlated positively with total quality of recovery (PRE Pearson 0.70, *p* < 0.05). And leptin correlated negatively with total quality of recovery (POST Pearson −0.75, *p* < 0.05) and vigor (PRE Pearson −0.85, *p* < 0.01) in finishers. In non-finishers, vigor correlated negatively with adiponectin (PRE Pearson −0.66, *p* < 0.01) and NPY (PRE Pearson −0.45, *p* < 0.05).

In non-finishers and the control group, vigor was positively correlated with cortisol (Non-finishers: D2 Pearson 0.71, *p* < 0.001; Control group: PRE Pearson 0.75, *p* < 0.05, D1 Pearson 0.70, *p* < 0.05). Also, vigor was positively correlated with perceived exertion in the control and non-finisher groups (Control group: D1 Pearson 0.74, *p* < 0.05; Non-finishers: D1 Pearson 0.54, *p* < 0.05, D2 Pearson 0.66, *p* < 0.01).

#### 4.5.5 Fatigue

At baseline, fatigue correlated negatively only in non-finishers with total quality of recovery (Pearson −0.61, *p* < 0.01). By contrast, during the race, it correlated positively with the total quality of recovery in the same group (D1 Pearson 0.47, *p* < 0.05, D2 Pearson 0.93, *p* < 0.001). At D1, fatigue correlated positively with adiponectin in finishers (Pearson 0.66, *p* < 0.05) and non-finishers (Pearson 0.50, *p* < 0.05). At D2, fatigue correlated negatively with adiponectin in the control group (Pearson −0.79, *p* < 0.05). In non-finishers and finishers, fatigue correlated positively with perceived exertion (Non-finishers: D1 Pearson 0.58, *p* < 0.01, D2 Pearson 0.89, *p* < 0.001; Finishers: D2 Pearson 0.72, *p* < 0.05, POST Pearson 0.63, *p* < 0.05), but not in the control group. This finding was highly significant in non-finishers at D2. Fatigue correlated positively with cortisol in the non-finisher and control groups (Non-finishers: D1 Pearson 0.46, *p* < 0.05, D2 Pearson 0.87, *p* < 0.001; Control group: D2 Pearson 0.77, *p* < 0.05). Fatigue correlated positively with confusion in non-finishers and finishers (Non-finishers: PRE Pearson 0.50, *p* < 0.05, D1 Pearson 0.77, *p* < 0.001, D2 Pearson 0.99, *p* < 0.001; Finishers: PRE Pearson 0.71, *p* < 0.05, D2 Pearson 0.85, *p* < 0.01).

#### 4.5.6 Confusion

Confusion correlated positively with tension-anxiety in non-finishers and finishers (Non-finishers: PRE Pearson 0.79, *p* < 0.01, D1 Pearson 0.76, *p* < 0.001, D2 Pearson 0.78, *p* < 0.001; Finishers: PRE Pearson 0.85, *p* < 0.01, D2 Pearson 0.78, *p* < 0.01). Whereas in the control group, only tension-anxiety, not fatigue, correlated positively with confusion (D1 Pearson 0.84, *p* < 0.05, D2 Pearson 0.97, *p* < 0.001).

In non-finishers, confusion correlated positively with perceived exertion (D1 Pearson 0.45, *p* < 0.05, D2 Pearson 0.94, *p* < 0.001). Also, confusion correlated positively with cortisol in non-finishers (D2 Pearson 0.91, *p* < 0.001). By contrast, only in finishers, confusion correlated negatively with cortisol (D2 Pearson −0.69, *p* < 0.05), adiponectin (POST Pearson −0.67, *p* < 0.05), and NPY (D1 Pearson −0.78, *p* < 0.01).

## 5 Discussion

This study examined the relationships between hormonal variables and mood states associated with participation in one of the coldest and longest ultramarathons in the world. The findings show that tension-anxiety scores decreased in finishers while tension-anxiety increased in non-finishers during the race. In both groups, finishers and non-finishers, fatigue scores increased. Noticeable was a decrease in vigor scores and an increase in confusion, anger, and depressive mood scores in non-finishers, not in the other groups. Leptin was associated with anger and a depressive mood state in non-finishers and worse recovery in finishers. In contrast, NPY appeared to be linked to reduced confusion and heightened quality of recovery in finishers.

Tharion et al. (1990) described that confusion significantly increased among athletes partaking in an ultramarathon. In the present study, the level of confusion was higher among all athletes than in the control group at baseline. This suggests an anticipatory affective state that was most likely a consequence of the race participants’ conscious thoughts about their circumstances before the race and the upcoming start. By contrast, increased levels of confusion seemed to be disadvantageous during the race. Only in non-finishers did confusion increase during the race and correlated positively with perceived exertion and cortisol. At the same time, cortisol contributed to a lower score of confusion in the finisher group. However, NPY also might have reduced finishers’ confusion scores during the race. Thus, these parameters notably appear to influence mood and probably contribute to the athletes’ endurance and success. It seems important how high a hormone or peptide rises and at which point of the race to achieve one of these effects on mood.

Lane et al. (2009) suggest that optimal performance depends on an interplay between emotional intelligence and mood states. They indicated that appraisal of own emotions was associated with low anger scores, and utilizing emotions was associated with low confusion and tension-anxiety scores (Lane et al., 2009). Among non-finishers, the level of anger increased during the race, while this effect did not occur among finishers. It is possible that finishers were more aware of their emotions perceived during the race and able to anticipate the potential effect of their emotions on performance. Non-finishers had higher depressive mood scores than the control group. In all three groups, the depressive mood score correlated positively with tension-anxiety and fatigue. Lane and Terry (2000) proposed a model suggesting that depressed mood is a moderating factor in the relationship between anger and tension-anxiety with performance. While anger and tension-anxiety can benefit performance when a depressed mood is absent, both are linked with poor performance when an athlete is experiencing a depressed mood. In their studies, Lane and Terry 2000; Lane, 2001 also showed that anger was associated with poor performance in a concentration grid test when sports students scored high on depressive mood. At the same time, tension-anxiety scores showed no significant relationship with performance in either group (Lane et al., 2001). Specifically, among non-finishers, depressive and anger mood scores increased during the race. Moreover, the depressive mood score correlated positively with anger in the non-finishers. This is in line with Lane and Terry’s observation (2000; 2001). There is evidence that an increase in these mood states - depression and anger - during racing can lead to failure in endurance sports.

Anger was associated with fatigue and tension-anxiety in all athletes, whether being finishers or non-finishers. In non-finishers, anger even correlated negatively with the quality of recovery. Tension-anxiety scores were significantly higher in all athletes than in the control group at baseline. Increased tension-anxiety scores in these athletes might be due to the high risks associated with failure and the loss of years of training and preparation. Since non-finishers scored higher on tension-anxiety, they might have perceived higher tension-anxiety than the finisher group during the first part of the race. The athletes described the first part of the route as particularly challenging and physically demanding. Tension-anxiety scores increased in non-finishers throughout the race, while they decreased in finishers. This contradicts one of the results of Lane and Terry (2000) and shows that poorer performance is related to an increase in tension-anxiety scores. The non-finishers seemed to struggle more with the race and their individual physical and psychological stress. This might have negatively influenced them in view of their own failure in this race. Graham et al. (2021) found that mental toughness correlated negatively with tension-anxiety during an Arctic ultramarathon. Tharion et al. (1990) compared finishers and non-finishers of an ultramarathon but did not find higher scores of tension-anxiety in one of these groups. The only significant difference between the groups was that finishers showed higher fatigue scores than non-finishers after the race (Tharion et al., 1990). In the present study, the fatigue score increased in all athletes more than in the control group. Similar to Tharion et al. (1990), no significant difference was found between non-finishers and finishers in fatigue scores during the race. In all athletes, tension-anxiety and fatigue scores correlated positively. It was interesting to note that perceived fatigue negatively affected recovery in non-finishers, while this was not the case in finishers. The finishers possibly found it easier to deal with fatigue without it harming recovery. They might have been mentally tougher. In non-finishers and the control group, vigor scores correlated positively with total quality of recovery. This correlation did not occur within the finishers. The finishers seemed not to depend on their vigor level to recover sufficiently. As has been shown in other studies, the level of vigor decreases during ultramarathon (Tharion et al., 1990; Lane and Wilson, 2011; Graham et al., 2021). Vigor decreased in both groups, finishers and non-finishers, but only in non-finishers was the decrease significant. This decline in vigor potentially affected the performance of non-finishers much more than finishers. Especially vigor is a crucial psychological attribute referring to physical strength, emotional energy, and cognitive liveliness (Shirom, 2011). Better performance seems to be associated with lower vigor scores, while poorer performance with higher confusion and depressive mood scores. Lane et al. (2009) revealed that optimism was associated with a high vigor score (Lane et al., 2009). In the present study, mainly fatigue scores increased during the race while vigor scores decreased in athletes. In other studies, similar effects of ultramarathons affecting mood states have been observed (Tharion et al., 1990; Graham et al., 2012; Rundfeldt et al., 2018; Graham et al., 2021). Slimani et al. (2018) showed that fatigue impairs body and cognitive performance in endurance athletes (Slimani et al., 2018). It is possible that both groups, finishers and non-finishers, are more optimistic than the control group. During the race, decreases in vigor probably affected optimism, but finishers handled the race better. Thus, they were more able to regulate their behavior to counteract negative mood states and emotions. Another study showed that vigor displayed a moderately strong correlation to sleep (Graham et al., 2012). Sleep is especially recognized as an essential component of recovery (Bird, 2013). In non-finishers and the control group, vigor correlated positively with the total quality of recovery, suggesting that vigor is also crucial for recovery. Furthermore, leptin correlated negatively with vigor and total quality of recovery in finishers, whereas NPY correlated positively with total quality of recovery. This shows the importance of these two transmitters in regulating effective recovery in athletes.

Brydon (2011) reported that higher basal leptin displayed greater stress-induced increases in heart rate and decreased heart rate variability (Brydon, 2011). Additionally, cortisol increases the level of leptin (Leal-Cerro et al., 2001). In the present study, we could not establish a relationship between cortisol and leptin. However, in this context, it is interesting to note that Rundfeldt et al. (2018) discovered an increased heart rate variability in finishers, indicating a reduced sympathetic activity. Thus, the finishers were less stressed than non-finishers. During a 25 km swim race, the level of NPY increased and corresponded with the successful completion of the competition (Karamouzis et al., 2002). Karamouzis et al. (2002) found that the increase in NPY was associated with a decrease in leptin. In the present study, leptin was negatively correlated with NPY. This finding occurred only in non-finishers and the control group.

Physical activity has been shown to reduce symptoms of anxiety and depression (Carek et al., 2011). Mainly running affects mood by reducing depression and confusion (Weinberg et al., 1988). In the present study, NPY appeared to decrease confusion scores in finishers, while leptin appeared to increase depressive mood scores. Morgan et al. (2000) showed that higher NPY levels were associated with improved higher performance under interrogation stress in Special Forces soldiers than non-Special Forces soldiers. The Special Forces soldiers’ group had a higher dissociation score if the level of NPY increased during interrogation (Morgan et al., 2000). In line with the present study, higher levels of NPY might help to cope with extremely challenging situations, as dissociation is part of our deeply ingrained survival system (Sar and Ross, 2006). Morgan et al. (2001; 2002) suggested that elevated NPY levels could improve self-confidence and performance, thus enhancing stress resistance. NPY might serve as a homeostatic buffer to attenuate stress responses. Genetic NPY alterations with a lower level of NPY are associated with PTSD (Yehuda et al., 2006; Sah et al., 2014) and major depression (Widerlöv et al., 1988; Sharma et al., 2022). Chang et al. (2016) showed that individuals with a genetic variant with high NPY expression had lower scores on anxiety and depression inventories during high stress than individuals with low NPY expression (Chang et al., 2016). Indeed, reduced NPY levels correlate with anxiety behaviors in patients with major depression (Widerlöv et al., 1988). Thus, NPY might reduce psychological distress and exerts anxiety-relieving effects. However, this effect could not be found within the correlation analysis. Instead, NPY slightly affected tension-anxiety scores in the non-finisher group at D2 by increasing them. However, at this measurement point, there was a chaos of feelings and transmitters in non-finishers, and no clear picture emerged.

Also, adiponectin affects mood by reducing depressive symptoms (Cao et al., 2018). But no negative correlation was found in the present study between the depressive mood score and adiponectin. The opposite effect emerged in non-finishers; adiponectin correlated positively with the depressive mood score. Roupas et al. (2013) evaluated the impact of an ultramarathon (180 km) on serum leptin and adiponectin. They could not find any changes in the adiponectin level but a significant decrease in leptin pre-vs. post-exercise (Roupas et al., 2013). Nevertheless, the exposition in the present study compared to that in Roupas’ study differs in several aspects. In Roupas et al. (2013), the distance was much shorter, and the race staff provided the participants with food and drinks. But the most important difference compared to the present study was certainly the weather conditions. The ultramarathon took place during the summer and not during cold winter conditions. In another ultramarathon study, adiponectin levels changed. Arakawa et al. (2016) showed that adiponectin levels increase in response to an ultramarathon (180 km) held during summer. While leptin levels first decreased and then increased after the race (Arakawa et al., 2016). This suggests that leptin and adiponectin regulation also depends on other factors besides temperature. In the control and non-finisher groups, adiponectin was related to leptin. Thus, leptin might have increased adiponectin or the other way around. But not in the finisher group; there was even no negative correlation between adiponectin and leptin. Zaccaria et al. (2002) examined the effects of three different endurance events on serum leptin concentrations in athletes: a half marathon (21.097 km), a ski mountaineering race (45 km), and an ultramarathon (100 km). The results suggest that only long-duration endurance exercises with high energy expenditure, such as alpine skiing and ultramarathon, cause a significant reduction in circulating serum leptin levels (Zaccaria et al., 2002). It takes much longer stretches for a change to occur at the hormonal and transmitter levels. Perhaps this is also dependent on the previous training sessions. It seems that from a certain point in extreme situations, a reduction of the leptin level is needed to survive or to cope with strenuous exercise. In non-finishers, leptin was associated with higher depressive mood scores. Clinical studies found elevated leptin levels in individuals with major depressive disorder (Milaneschi et al., 2017). Increases in leptin are associated with starchy food-seeking (Licinio et al., 2014). In contrast, decreased leptin levels and increased NPY levels activate feeding behavior by stimulating the seeking and finding of food (Woods et al., 1998; Licinio et al., 2014), which might be an important evolutionary motivational effect for athletes in such extreme ultramarathons.

In conclusion, the study reveals an essential interplay of hormones and peptides affecting mood states during endurance exercise: Leptin appears to be connected with depressive mood and anger scores, and decreases vigor scores. NPY seems to reduce confusion scores and enhances the quality of recovery. It has also been shown that some mood states are conditional on others: depressive mood might cause confusion, anger, and tension-anxiety. Confusion might cause fatigue and tension-anxiety, while increasing perceived exertion. Vigor might improve the quality of recovery.

However, it should be remembered that this is a small and hard-to-reach population, and conducting a study under such extreme conditions is likely to remain a challenge.

## 6 Limitations

The race distance limits the present study results because the observed changes in physiological markers of exercise might vary in shorter or longer distances. The race under examination is one of the longest and coldest ultramarathons worldwide. In order to achieve a decent number of participants, the athletes from 2015, 2017, and 2019 were pooled. Thus, caution is needed to generalize the findings to other distances and climate conditions. Furthermore, a limitation of the present study was that it had a pre-experimental design. Participants would compete in cold and normal temperatures in a true experimental model, i.e., a randomized-groups model. However, such an optimal design was not feasible. The study is about changes in mood states and not about the diagnosis of depression in ultramarathon runners. The authors would like to point out that the POMS-SF is a questionnaire, not a clinical diagnostic tool.

## Data Availability

The original contributions presented in the study are included in the article/Supplementary Material; further inquiries can be directed to the corresponding author.

## Supplementary material

Supplementary Figure S1 POMS-SF scores at the four different time points and the three groups: finisher (FIN, dark grey), non-finisher (NON, middle grey dotted line), and control (CON, light grey dotted line).

## Abbreviations

BIA, bioelectrical impedance analysis; BMI, body mass index; NPY, Neuropeptide Y; POMS, Profile of Mood States; SF, Short Form; YAU, Yukon Arctic Ultra.
