# Supplementary material for: Adiponectin, leptin, cortisol, neuropeptide Y and profile of mood states in athletes participating in an ultramarathon during winter: An observational study
Source: Front Physiol. 2022 Dec 12;13:970016. doi: 10.3389/fphys.2022.970016 (PMC9791263; doi:10.3389/fphys.2022.970016)
Supplement: Supplementary file 1 [file Table1.docx]

| **Time points** | |  | **PRE** | | **D1** | | **D2** | | **POST** | |
| --- | --- | --- | --- | --- | --- | --- | --- | --- | --- | --- |
| **Group** | **Gender** | **n** | **Leptin, ng/ml** | | **Leptin, ng/ml** | | **Leptin, ng/ml** | | **Leptin, ng/ml** | |
|  |  |  |  | m ± SD |  | m ± SD |  | m ± SD |  | m ± SD |
| *FIN* | Men | 4 | 3.75 | 4.91 | 1.15 | 0.30 | 1.90 | 1.07 | 1.10 | 0.20 |
|  | Woman | 5 | 4.72 | 3.67 | 2.76 | 1.77 | 2.50 | 1.50 | 1.58 | 0.80 |
|  | All | 9 | 4.29 | 4.00 | 2.04 | 1.52 | 2.23 | 1.29 | 1.37 | 0.63 |
| *NON* | Men | 13 | 2.98 | 1.93 | 1.15 | 0.30 | 2.30 | NA | NaN | NA |
|  | Woman | 7 | 14.08 | 15.57 | 2.76 | 1.77 | 1.00 | NA | NaN | NA |
|  | All | 20 | 6.15 | 9.25 | 4.36 | 8.80 | 1.65 | 0.92 | NaN | NA |
| *CON* | Men | 2 | 2.30 | NA | 1.50 | NA | 1.00 | 0.00 | 1.10 | NA |
|  | Woman | 5 | 10.73 | 5.29 | 10.80 | 6.20 | 8.40 | 5.39 | 14.40 | 4.32 |
|  | All | 7 | 9.04 | 5.93 | 8.48 | 6.88 | 5.44 | 5.56 | 11.74 | 7.02 |
| *ALL* | Men | 19 | 3.14 | 2.78 | 1.18 | 0.33 | 0.91 | 0.91 | 1.10 | 0.17 |
|  | Woman | 17 | 9.45 | 9.44 | 7.14 | 8.36 | 4.30 | 4.25 | 7.28 | 7.28 |
|  | All | 36 | 6.07 | 7.34 | 4.16 | 6.53 | 3.16 | 3.43 | 5.07 | 6.48 |

**Supplementary Table 1:** *Leptin (ng/ml) levels at the four different time points and in the three groups. FIN = Finisher, NON = Non-Finisher, CON = Control group, m = mean, SD = Standard Deviation.*
