# Supplementary figures and images for: Adiponectin, leptin, cortisol, neuropeptide Y and profile of mood states in athletes participating in an ultramarathon during winter: An observational study
Source: Front Physiol. 2022 Dec 12;13:970016. doi: 10.3389/fphys.2022.970016 (PMC9791263; doi:10.3389/fphys.2022.970016)

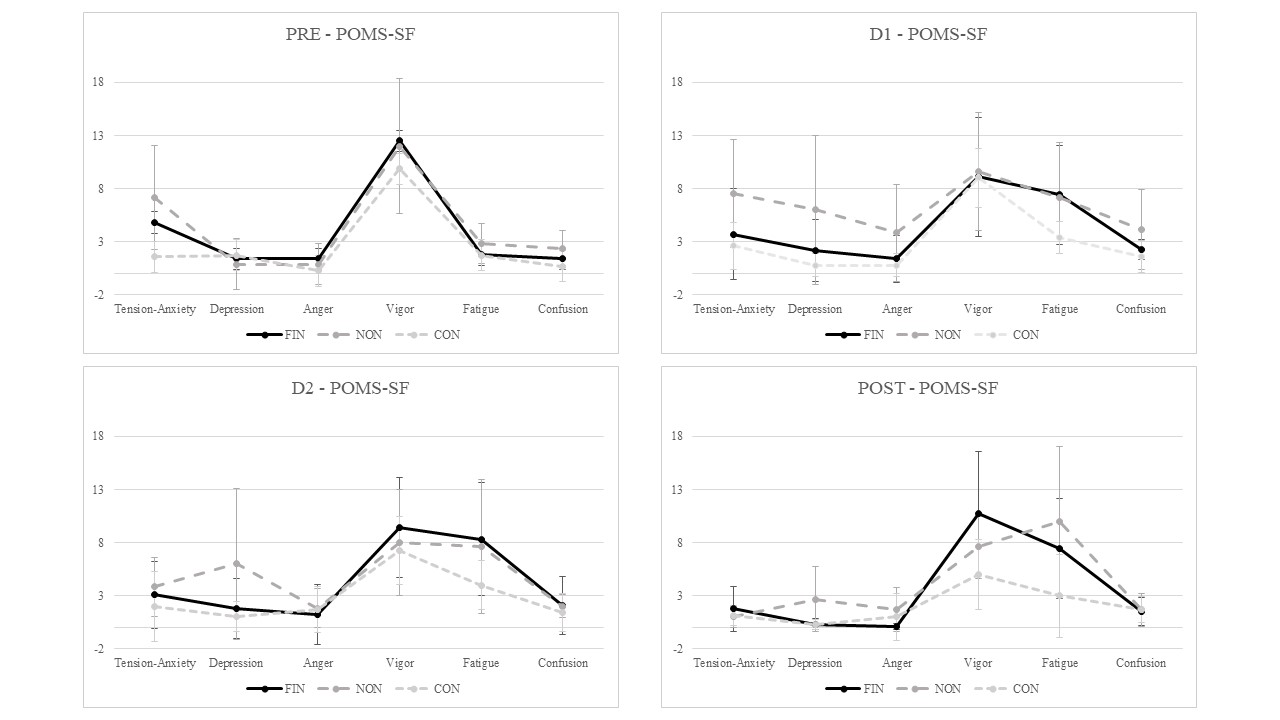

Supplement: Supplementary file 2 [file Image1.JPEG]
