# Supplementary material for: Adiponectin, leptin, cortisol, neuropeptide Y and profile of mood states in athletes participating in an ultramarathon during winter: An observational study
Source: Front Physiol. 2022 Dec 12;13:970016. doi: 10.3389/fphys.2022.970016 (PMC9791263; doi:10.3389/fphys.2022.970016)
Supplement: Supplementary file 3 [file Table2.docx]

| **Time points** | |  | **PRE** | | **D1** | | **D2** | | **POST** | |
| --- | --- | --- | --- | --- | --- | --- | --- | --- | --- | --- |
| **Group** | **Gender** | **n** | **NPY, pg/ml** | | **NPY, pg/ml** | | **NPY, pg/ml** | | **NPY, pg/ml** | |
|  |  |  |  | m ± SD |  | m ± SD |  | m ± SD |  | m ± SD |
| *FIN* | Men | 4 | 7.92 | 2.87 | 16.31 | 10.17 | 13.80 | 7.98 | 8.65 | 7.25 |
|  | Woman | 5 | 9.20 | 7.21 | 11.51 | 7.01 | 10.30 | 3.57 | 5.25 | 2.50 |
|  | All | 9 | 8.63 | 5.43 | 14.25 | 8.64 | 11.85 | 5.80 | 6.76 | 5.10 |
| *NON* | Men | 13 | 4.90 | 2.12 | 15.25 | 5.90 | 21.40 | NA | 8.03 | NA |
|  | Woman | 7 | 8.78 | 6.52 | 9.48 | 7.46 | 12.33 | NA | NaN | NA |
|  | All | 20 | 7.57 | 5.75 | 13.32 | 6.63 | 16.87 | 6.41 | 8.03 | NA |
| *CON* | Men | 2 | 6.51 | 4.24 | 20.56 | NA | 7.05 | 5.95 | 7.08 | 0.78 |
|  | Woman | 5 | 16.46 | 11.28 | 12.70 | 5.45 | 9.08 | 4.49 | 9.84 | 7.77 |
|  | All | 7 | 12.48 | 9.89 | 14.67 | 5.93 | 8.40 | 4.50 | 9.05 | 6.49 |
| *ALL* | Men | 19 | 8.31 | 5.47 | 16.11 | 7.13 | 12.96 | 7.86 | 8.11 | 5.19 |
|  | Woman | 17 | 9.22 | 7.80 | 11.23 | 5.97 | 10.02 | 3.66 | 7.54 | 5.96 |
|  | All | 36 | 8.70 | 6.47 | 13.92 | 6.93 | 11.23 | 5.74 | 7.78 | 5.49 |

**Supplementary Table 2:** *Neuropeptide Y (NPY) (pg/ml) levels at the four different time points and in the three groups. FIN = Finisher, NON = Non-finisher, CON = Control group, m = mean, SD = Standard Deviation.*
