# Supplementary material for: Adiponectin, leptin, cortisol, neuropeptide Y and profile of mood states in athletes participating in an ultramarathon during winter: An observational study
Source: Front Physiol. 2022 Dec 12;13:970016. doi: 10.3389/fphys.2022.970016 (PMC9791263; doi:10.3389/fphys.2022.970016)
Supplement: Supplementary file 4 [file Table3.docx]

| **Time points** | |  | **PRE** | | **D1** | | **D2** | | **POST** | |
| --- | --- | --- | --- | --- | --- | --- | --- | --- | --- | --- |
| **Group** | **Gender** | **n** | **Adiponectin, µg/ml** | | **Adiponectin, µg/ml** | | **Adiponectin, µg/ml** | | **Adiponectin, µg/ml** | |
|  |  |  |  | m ± SD |  | m ± SD |  | m ± SD |  | m ± SD |
| *FIN* | Men | 4 | 7.60 | 7.21 | 14.70 | 10.42 | 16.67 | 13.63 | 21.35 | 10.87 |
|  | Woman | 5 | 9.95 | 3.66 | 21.02 | 11.03 | 22.14 | 10.60 | 22.86 | 4.55 |
|  | All | 9 | 8.94 | 5.06 | 18.65 | 10.55 | 20.09 | 11.19 | 22.19 | 7.44 |
| *NON* | Men | 13 | 8.82 | 5.08 | 11.88 | 4.27 | 9.90 | NA | NaN | NA |
|  | Woman | 7 | 11.88 | 3.64 | 14.73 | 5.58 | 23.90 | NA | NaN | NA |
|  | All | 20 | 9.69 | 4.80 | 12.83 | 4.60 | 16.90 | 9.90 | NaN | NA |
| *CON* | Men | 2 | 4.40 | NA | 5.20 | NA | 4.30 | 0.57 | 4.00 | NA |
|  | Woman | 5 | 10.85 | 2.36 | 16.67 | 7.22 | 16.73 | 9.58 | 11.33 | 1.07 |
|  | All | 7 | 9.56 | 3.53 | 13.80 | 8.22 | 11.76 | 9.61 | 9.86 | 3.40 |
| *ALL* | Men | 19 | 8.24 | 5.23 | 12.06 | 6.46 | 11.42 | 10.57 | 17.88 | 12.20 |
|  | Woman | 17 | 10.89 | 3.08 | 18.12 | 8.58 | 20.53 | 9.36 | 17.73 | 6.91 |
|  | All | 36 | 9.47 | 4.50 | 15.23 | 8.08 | 16.89 | 10.55 | 17.79 | 8.67 |

**Supplementary Table 3:** *Adiponectin (µg/ml) levels at the four different time points and in the three groups. FIN = Finisher, NON = Non-finisher, CON = Control group, m = mean, SD = Standard Deviation.*
