# Supplementary material for: Adiponectin, leptin, cortisol, neuropeptide Y and profile of mood states in athletes participating in an ultramarathon during winter: An observational study
Source: Front Physiol. 2022 Dec 12;13:970016. doi: 10.3389/fphys.2022.970016 (PMC9791263; doi:10.3389/fphys.2022.970016)
Supplement: Supplementary file 5 [file Table4.docx]

| **Time points** | |  | **PRE** | | **D1** | | **D2** | | **POST** | |
| --- | --- | --- | --- | --- | --- | --- | --- | --- | --- | --- |
| **Group** | **Gender** | **n** | **Cortisol, µg/ml** | | **Cortisol, µg/ml** | | **Cortisol, µg/ml** | | **Cortisol, µg/ml** | |
|  |  |  | **m ± SD** | | **m ± SD** | | **m ± SD** | | **m ± SD** | |
| *FIN* | Men | 4 | 24.24 | 19.30 | 22.94 | 0.75 | 21.08 | 7.89 | 19.70 | 4.92 |
|  | Woman | 5 | 16.03 | 4.29 | 21.34 | 11.46 | 20.35 | 4.95 | 20.29 | 11.44 |
|  | All | 9 | 20.13 | 13.87 | 22.05 | 8.16 | 20.78 | 5.14 | 20.00 | 8.31 |
| *NON* | Men | 13 | 20.61 | 9.19 | 15.65 | 6.26 | 18.77 | 4.64 | 23.40 | 5.09 |
|  | Woman | 7 | 22.39 | 12.58 | 9.12 | 2.10 | 23.80 | 5.66 | 37.40 | NA |
|  | All | 20 | 21.26 | 10.26 | 12.68 | 5.74 | 20.72 | 6.23 | 28.07 | 8.85 |
| *CON* | Men | 2 | 11.35 | 4.03 | 15.95 | 5.30 | 20.30 | 0.14 | 20.25 | 2.33 |
|  | Woman | 5 | 11.46 | 14.01 | 6.67 | 6.33 | 13.92 | 12.60 | 18.68 | 7.28 |
|  | All | 7 | 11.43 | 11.55 | 10.38 | 7.27 | 15.74 | 10.75 | 19.13 | 6.07 |
| *ALL* | Men | 19 | 20.59 | 12.19 | 18.13 | 5.76 | 20.23 | 5.80 | 20.65 | 4.30 |
|  | Woman | 17 | 17.30 | 11.64 | 13.26 | 9.86 | 18.25 | 9.26 | 21.12 | 10.17 |
|  | All | 36 | 19.04 | 11.88 | 15.59 | 8.36 | 19.15 | 7.77 | 20.90 | 7.89 |

**Supplementary Table 4:** *Cortisol* *(µg/ml) levels at the four different time points and in the three groups. FIN = Finisher, NON = Non-finisher, CON = Control group, m = mean, SD = Standard Deviation.*
